# Rheumatic Fever Associated with Antiphospholipid Syndrome: Systematic Review

**DOI:** 10.1155/2014/614591

**Published:** 2014-04-22

**Authors:** Felipe da Silva, Jozélio de Carvalho

**Affiliations:** ^1^Escola Bahiana de Medicina e Saúde Pública, 41810080 Salvador, BA, Brazil; ^2^Rheumatology Division, Centro Médico do Hospital Aliança, Rua das Violetas 42, AP. 502, 41810080 Salvador, BA, Brazil

## Abstract

*Objective*. To evaluate the clinical associations between rheumatic fever and antiphospholipid syndrome and the impact of coexistence of these two diseases in an individual. *Methods*. Systematic review in electronics databases, regarding the period from 1983 to 2012. The keywords: “Rheumatic Fever,” “Antiphospholipid Syndrome,” and “Antiphospholipid Antibody Syndrome” are used. *Results*. were identified 11 cases described in the literature about the association of rheumatic fever and antiphospholipid syndrome. Clinical presentation of rheumatic fever was characterized by the predominance of carditis (11/11) and chorea (7/11). Regarding the manifestations of APS, the stroke was observed in 7/11 (63.6%), with one of them having probable embolic origin. *Conclusion*. The present study brings the information that the association between APS and RF is quite rare, however, is of great clinical importance. Doctors who deal with the RF should include in their differential diagnosis the APS, especially in the presence of stroke in patients with RF and whose echocardiogram does not show intracavitary thrombi.

## 1. Introduction

Rheumatic fever (RF) is a nonsuppurative late autoimmune inflammatory complication of* Streptococcus pyogenes* infection in the upper airway. This condition remains a public health problem in developing countries. The clinical picture consists mainly of episodes of migratory polyarthritis, Sydenham's chorea, and rheumatic heart disease [1].

Antiphospholipid syndrome (APS) is an autoimmune disease characterized by thrombotic events and/or recurrent abortions in the presence of antiphospholipid antibodies (aPLs) [2]. RF and APS share clinical and pathophysiological manifestations, among which we highlight the carditis, chorea, arthritis, and thromboembolic events (mainly represented by stroke). Furthermore, aPLs have been related to valve manifestations which are similar in both pathologies, as much from the standpoint echocardiographic as pathophysiological [3] (this paper showed a considerable overlap of humoral immunity in RF and APS, supporting the hypothesis of common pathogenic mechanisms between both diseases). Previous studies also were able to demonstrate that aPLs are associated with the onset of chorea in APS [4, 5].

On the other hand, when the scientific literature is analyzed, the relationship between APS and RF has been little studied, and there is no article which conducted a review about this association. Therefore, the aim of this study was to evaluate the clinical associations between rheumatic fever and antiphospholipid syndrome and the impact of coexistence of these two diseases in an individual.

## 2. Methods

This is a systematic review of scientific papers that deal with the relationship between rheumatic fever and antiphospholipid syndrome. A search was performed in electronic databases: PubMed, MEDLINE, LILACS, SciELO, and Cochrane Database, regarding the period from 1983 (when the antiphospholipid syndrome was described) to October 2012. A combination of the keywords “Rheumatic Fever,” “Antiphospholipid Syndrome,” “Antiphospholipid Antibody Syndrome,” and their respective translations was used. Articles in English, Portuguese, and Spanish were considered. There was no restriction about the study design.

The combination search of keywords was performed by two authors independently and showed 22 scientific articles. A full reading of each of these papers and their references reduced the universe of work for 11 articles that evaluated the relationship of antiphospholipid syndrome with rheumatic fever. Of these, only 7 showed clinical cases of association between APS and RF, being composed of 2 cross-sectional studies and 5 case reports. However, we have used only one cross sectional study since they describe the same patients.

## 3. Results

Therefore 11 cases described in the literature about the association of rheumatic fever and antiphospholipid syndrome were identified [6–11].

The demographic, clinical, and laboratorial data of patients are summarized in Table 1.

The age of patients with APS and RF ranged from 22 to 45 years, with a predominance of females (10/11 patients) and white (6/7). The duration of illness ranged to APS from 5.6 to 24 years and for rheumatic fever from 1.5 to 35 years.

Clinical presentation of rheumatic fever was characterized by the predominance of carditis (11/11) and chorea (7/11). Arthritis was observed in 3 of 11 patients and only one individual presented subcutaneous nodule (9%). No patient had erythema marginatum. Serum levels of ASO (antistreptolysin-O) were elevated in most cases (9/11), with values ranging from 451 to 883 UI/m. In patients in whom it was studied for antiDNAse, this antibody was also positive and with high titers. The prophylaxis for rheumatic fever was reported in all cases, being the benzathine penicillin used as the first choice (8/11), followed by sulfadiazine (1/11) and oral penicillin (1/11).

Regarding the manifestations of APS, stroke was observed in 7/11 (63.6%), being one of them from probable embolic origin. Three of eleven (27.3%) patients had venous event characterized by deep vein thrombosis (DVT). Thrombocytopenia was observed in 3 of 11 cases and no article described the presence of livedo reticularis. Abortion was described in only 1 of 10 individuals. Acute myocardial infarction (AMI) was present in 2 of 10 patients, also being reported the occurrence of splenic infarction in one patient. The antibodies research showed that anticardiolipin (aCL) IgM was the most found antibody and was positive in 5/7 patients, followed by anti-*β*2GPI in 4/6 and lupus anticoagulant (LAC) in 5/8. Already the aCL IgG antibody was present in 3/8.

In only two cases manifestations of systemic lupus erythematosus (SLE) were presented, characterized by pericarditis and positive antibodies (ANA and anti-dsDNA). In two of the patients the presence of anti-dsDNA (antidouble stranded DNA) without other manifestations of SLE and other presented positive ANA (antinuclear antibody) was reported singly. There was no report of another autoimmune disease, besides RF, APS, or SLE.

Common clinical manifestations of APS and RF were distributed as follows: carditis with valvular disease in 100% of cases, chorea in 63.6%, and arthritis in 18.2%.

## 4. Discussion

The present work performed the first systematic review of all clinical cases published in the literature which presented an association between APS and RF.

The major advantage of the present study was carrying out an extensive and complete literature review, for the first time, on the main world databases (PubMed, MEDLINE, LILACS, SciELO, and Cochrane) regarding the period of 1983 to October 2012, with the total of 11 cases of association between APS and RF.

In the scientific literature, there is some evidence of the relationship between APS and RF. In fact, the clinical and experimental study published by Blank et al., which evaluated the serum of 90 patients with RF and of 42 patients with APS, showed that 24% of patients with rheumatic heart disease had positivity for anti-*β*2GPI (antibeta-2-glycoprotein I) and that patients with APS had antistreptococcal activity, recognizing the M protein in 16.6% of cases. Interestingly, this study concluded that there is some overlap in the activity of anti-*β*2GPI and anti-M protein/N-acetylglucosamine, suggesting common mechanisms in development of heart valve and neurological manifestations in APS and RF [3]. The same authors, in a previous study, had also shown a possible infectious origin of the SAF, where several infectious agents, including* Streptococcus pyogenes*, are able to induce the formation of aPLs, responsible for the clinical manifestations of this condition [12] (this work showed a possible infectious origin of the SAF, where several infectious agents, including* Streptococcus pyogenes*, are able to induce the formation of antiphospholipid antibodies).

The study by Figueroa et al. showed that during the active phase of FR, 80% of patients had positive anticardiolipin antibodies (aCL). On the other hand, during the inactive phase of the disease, only 40% of these patients had such antibodies [13] (this study showed that 80% of RF patients were positive for anticardiolipin antibodies during active phase of the disease). In the same line of study, Carvalho and Goldenstein-Schainberg simultaneously evaluated the presence of three antiphospholipid antibodies routinely used (aCL, lupus anticoagulant, and anti-*β*2GPI) in a group of nine patients with long-term FR (about eleven years). In contrast to other studies, no patient showed positivity to any of these antibodies [14].

This systematic review has highlighted that all patients with APS and RF had carditis, constituting the most frequent manifestation. In fact, the literature indicates that about 30–45% of patients with RF develop carditis, its most serious complication, responsible for permanent valvular lesions [15] (this paper highlights a possible link between the mechanisms of heart afflictions in RF and APS). In the places of cardiac injury, a predominance of CD4 + T cells and macrophages exists, besides deposition of complement and expression of VCAM-1 (vascular cell adhesion protein 1) in the endothelium valve. Histologically, cardiac injury can be characterized by Aschoff's nodes [15]. Cardiac involvement can also be explained by cross-reactivity of anti-M protein with myocardial proteins such as myosin, tropomyosin, and protein valvular endocarditis triggering feature of FR [3]. The valve involvement in APS affects about 32–38% of patients and is associated with high levels of antiphospholipids antibodies [15]. The cardiovascular disorder in this syndrome occurs due to deposition of immune complexes, which may lead to formation of vegetations and valvular dysfunction, characterized by thickening of the valve leaflets and getting the name of aseptic endocarditis Libman-Sacks [16]. The mitral valve is the most affected, followed by the aortic and tricuspid valves. Intracardiac thrombi are rare but represent high rate of morbimortality [16]. Therefore, although known cross-reactivity between molecules of *β*2GPI and M-protein that can contribute to heart damage, APS and FR have different pathogenic mechanisms with regard to the valve involvement [15].

Regarding chorea, whose prevalence in the APS is around 1.3% [17], this study found that 63.6% of our patients had APS associated chorea. This neurological manifestation has been evaluated in a few studies in the literature, although, in the same series, patients with SLE or other autoimmune diseases were included [17]. In order to evaluate chorea in a group of patients with primary APS, Appenzeller et al. studied 88 patients with APS and identified 4 patients with chorea. Interestingly, the presence of chorea in patients with APS was associated with RF and thrombocytopenia [18] (this study suggests a link between APS patients presented with chorea and rheumatic fever), suggesting the need for echocardiography (ECHO) in all patients with APS and chorea. The presence of chorea may be explained from the same mechanisms proposed for rheumatic carditis, when occurs cross reactivity against self-antigen (basal ganglia cells) and thus generating the characteristic movement disorder [3]. Moreover, antiphospholipid antibodies have also been associated with chorea, both in APS and in RF and SLE [11].

One feature that caught the attention in APS manifestations of this study was the prevalence of stroke, present in 63.6% of cases. This finding is confirmed in the literature by the work of Camargo et al., where they compared 5 patients with APS associated with RF with 68 patients presented with primary APS. Remarkably, the presence of stroke was significantly higher in the first group (80% versus 25%); however, the echocardiogram in these patients showed no intracardiac thrombi. These results suggest a possible formation of thrombus in situ [7] (this paper suggests that APS patients associated with RF have higher stroke risk when compared with primary APS patients).

The achievement of this paper has made the design of the profile of a patient with APS and RF possible. This individual is typically female, white, and aged between 22 and 45 years. The duration of disease in this patient is around 5 years for APS and can vary from 1.5 to 35 for RF, and usually the FR appears as the first manifestation. In relation to clinical manifestations of RF, these individuals will follow the pattern of involvement of this condition; in other words, they will have more carditis and chorea with high ASO and will receive antibiotic prophylaxis. However, for the manifestations of APS, interestingly, arterial thrombosis was more common than vein thrombosis, as discussed above. DVT, as main venous event of this syndrome, which afflicts about 55% of patients [19], occurred in only 27.3% of the subjects in our study. No description of the presence of livedo reticularis was reported, although it is the most common cutaneous manifestation of APS [20]. Regarding antibodies, the most commonly found are the aCL IgM followed by anti-*β*2GPI.

This study provides information that the association between SAF and FR is quite rare, however, is of great clinical importance because the isolated treatment of one of these diseases, such as lack of anticoagulation in an individual with APS hidden and lack of prophylaxis of streptococcal infection in an individual with RF undiagnosed, can lead to serious consequences. This study also warns the doctors who deal with the RF, condition still very common in our environment, such as rheumatologists, pediatricians, cardiologists, cardiac surgeons, and general practitioner, to include in their differential diagnosis the APS, especially in the presence of stroke in patients with RF and whose echocardiogram did not show intracavitary thrombi.

## Key Issues

- The relationship between APS and RF has been little studied.
- 11 cases described in the literature about the association of rheumatic fever and antiphospholipid syndrome were identified.
- Common clinical manifestations of APS and RF were distributed as follows: carditis with valvular disease in 100% of cases, chorea in 63.6%, and arthritis in 18.2%.
- Previous study showed that 24% of patients with rheumatic heart disease had positivity for anti-*β*2GP and that patients with APS had antistreptococcal activity, recognizing the M protein in 16.6% of cases.
- This systematic review has highlighted that all patients with APS and RF had carditis, constituting the most frequent manifestation.
- Regarding chorea, whose prevalence in the APS is around 1.3% [17], this study found that 63.6% of our patients had APS associated chorea.
- About APS manifestations, interestingly, arterial thrombosis was more common than vein thrombosis.
- This study provides information that the association between SAF and FR is quite rare, however, is of great clinical importance.
- This study warns clinicians to include in their differential diagnosis the APS, especially in the presence of stroke in patients with RF and whose echocardiogram did not show intracavitary thrombi.

## Figures and Tables

**Table 1:** 11 cases published in the literature about the association between antiphospholipid syndrome and rheumatic fever.

References	Number of cases	Age, years	Female sex	Color	Disease duration, years	RF manifestations	APS manifestations	SLE associated	ASO	Antiphospholipid antibody	RF prophylaxis
Soeiro et al., 2012 [6]	1	30	100%	Brown	1.5 RF-APS	Carditis: + (with mitral valve replacement, right bundle branch block) Chorea: + Arthritis: −	Arterial: embolic stroke, AMI Venous: − Obstetric: − Thrombocytopenia: + Livedo: − Chorea: +	Yes	786 UI/mL	aCL IgM: + (14 MPL)	Penicillin G benzathine 100%

Camargo et al., 2012 [7]	73 APS, 5 RF-APS	37.4	100%	100% white	5.6 RF 5.6 APS	Carditis (ECO abnormal): 100% Chorea: 2/5 Arthritis: −	Arterial: stroke 4/5 Venous: DVT 2/5 Obstetric: − Thrombocytopenia: 1/5 Livedo: − Chorea: 2/5	No	Increased in acute phase: 100%	aCL IgG: 2/5 aCL IgM: 3/5 LAC: 2/5 Anti-*β*2GPI(IgG): 3/5	Penicillin G benzathine 100%

Alcock et al., 2011 [8]	1	44	100%	—	27 RF 24 APS	Carditis: + (ECG: block first degree ECO: mitral thickening) Chorea: + Arthritis: +	Arterial: stroke Venous: − Obstetric: abortion, fetal and maternal hypertension Thrombocytopenia: + Livedo: − Chorea: +	No	883 UI/mL, Anti-DNAase B: 960 UI/mL	aCL: + LAC: + anti-*β*2GPI (IgG): 117 SGU/mL	Penicillin 100%

Izhevsky et al., 2004 [9]	1	29	0%	—	-RF9 APS	Carditis: + (block AV first and third grades) Chorea: − Arthritis: + Subcutaneous nodule: +	Arterial: AMI (with CRA and successful resuscitation) Venous: DVT Thrombocytopenia: − Livedo: − Chorea: −	No	Increased	aCL IgG: + LAC: +	Penicillin G benzathine 100%

Amital et al., 1999 [10]	1	45	100%	—	35 RF 12 APS	Carditis: + (admitted for mitral valve replacement) Chorea: + Arthritis: −	Arterial: stroke, splenic infarction Venous: − Obstetric: − Thrombocytopenia: − Livedo: − Chorea: +	Yes	—	LAC: +	Prophylaxis with antibiotic

Fung et al., 1998 [11]	2	Case 1: 22 Case 2: 31	100%	1 white, 1 not described	Case 1: − Case 2: 15 RF 2 APS	Case 1: Carditis: + (ECO: rheumatic valve disease, systolic murmur) Chorea: + Arthritis: − Case 2: Carditis: + (mitral murmur) Chorea: + Arthralgia: +	Cases 1 and 2: Arterial: − Venous: − Obstetric: − Thrombocytopenia: − Livedo: − Chorea: +	Case 1: anti-dsDNA positive (35%) Case 2: ANA positive	Case 1: normal, anti-DNAase B: 1280 UI/mL Case 2: 451 UI/mL	Case 1: aCL IgM: + (14 MPL) Case 2: aCL: +	Case 1: oral penicillin Case 2: sulfadiazine

AMI: acute myocardial infarction; AV: atrioventricular; CRA: cardiorespiratory arrest; DVT: deep vein thrombosis; ECG: electrocardiogram; ECO: echocardiogram.
